# Survey analysis and discussion on cultivating scientific research quality among undergraduates in medical colleges

**DOI:** 10.1002/prp2.1095

**Published:** 2023-05-19

**Authors:** Ling Liu, Jing Luan

**Affiliations:** ^1^ Biomedical Sciences College & Shandong Medicinal Biotechnology Centre Shandong First Medical University & Shandong Academy of Medical Sciences Ji'nan China; ^2^ The First Affiliated Hospital of Shandong First Medical University, Key Lab for Biotechnology Drugs of National Health Commission, Key Lab for Rare & Uncommon Diseases of Shandong Province, Biomedical Sciences College & Shandong Medicinal Biotechnology Centre Shandong First Medical University & Shandong Academy of Medical Sciences Ji'nan China

**Keywords:** medical students, questionnaire survey, scientific research quality, undergraduates

## Abstract

To explore rational measures to improve medical undergraduates' scientific research quality by investigating and analyzing their scientific research situation. A questionnaire survey was conducted in March 2022 among medical college/university undergraduates across four grades and five majors. Five hundred and ninety‐four questionnaires were distributed, and 553 valid copies were returned, with a 93.1% return rate. The results showed that 61.5% of the students had an intense interest in research experiments, and 46.8% thought it was important for undergraduates to participate in research experiments, but only 17.5% often participated in them. Among the students, 85.0% thought that the main factors preventing them from participating in research experiments were academic stress and insufficient time, and 82.6% hoped that mentors would focus on practical skills training; only 13.0% read literature at least once per week, and 93.5% were not proficient at organizing and using literature. Among the participating undergraduates, more than half were strongly interested in scientific research, but academic stress, unclear participation modes, and insufficient literature retrieval skills limited undergraduate scientific research practice and improvement of scientific quality. Therefore, it is essential to cultivate undergraduates' interest in scientific research, ensure that they have spare time to engage in scientific research, improve the undergraduate scientific research mentorship system, and enhance relevant scientific research abilities to cultivate more innovative talent in scientific research.

**Abbreviation**
CET 4 and 6, college english test band 4 and band 6

## 1 INTRODUCTION

Cultivating undergraduate scientific research quality is a major step in the cultivation of innovative talent. Entrusted with the responsibility of cultivating students' scientific research and innovation ability, colleges and universities in China implemented many successive training programs with such an aim at the end of the last century to reinforce the cultivation of undergraduate scientific research quality.^1^ As early as November 2006, the Ministry of Education launched the National Innovative Experimentation Program for Undergraduates, which advocated innovative experimentation reform for undergraduates, encouraged students to participate in scientific research and invention training at the undergraduate stage, and promoted colleges' and universities' exploration and establishment of a problem‐ and project‐based teaching model to provide more platforms for the cultivation of undergraduate scientific research quality.^1^ Moreover, the Challenge Cup, a national college students’ extracurricular academic science and technology works competition, has been held every 2 years since 1989 to facilitate the development of extracurricular academic and scientific activities among college students. This effort has resulted in the discovery and fostering of considerable outstanding talent with academic potential in science and technology. Medical colleges' and universities' scientific research development is particularly prominent. Currently, with an emphasis on internationalization, platforms, and innovation, medical colleges and universities in China are introducing high‐end talent, acquiring large instruments and equipment at an internationally competitive level of advancement, and cultivating innovative talent with high scientific research quality, as these institutions strive to build a first‐class scientific research platform. However, some phenomena have recently emerged in the cultivation of undergraduate scientific research quality in medical colleges and universities in China, such as students' disinterest in scientific research, insufficient attention among schools and teachers to undergraduates' research experiments,^2^, ^3^ and insufficient exercise of students' research ability.^4^ These problems seriously hinder colleges' and universities' efforts to foster innovative talent in scientific research. Therefore, this study identified the prominent problems via questionnaires to examine viable paths for cultivating undergraduates' research quality, actively investigate effective methods of stimulating undergraduates' desire for research exploration, expand the modes of research skills training, and reinforce research concepts, to refine the innovative talent cultivation system.

## 2 RESEARCH PARTICIPANTS AND METHODS

### 2.1 Research participants

Among the 553 research subjects, 202 students were male (36.5%), and 351 were female (63.5%), majoring in biotechnology (315 students; 57.0%), pharmacy (96 students; 17.3%), clinical medicine (76 students; 13.7%), medical insurance (35 students; 6.3%), and laboratory animals (31 students; 6.0%). Regarding the year of study, 208 (37.6%) were freshmen, 215 (38.9%) were sophomores, 79 (14.3%) were juniors, and 51 were (9.2%) seniors.

### 2.2 Survey content and methods

**FIGURE 1:**
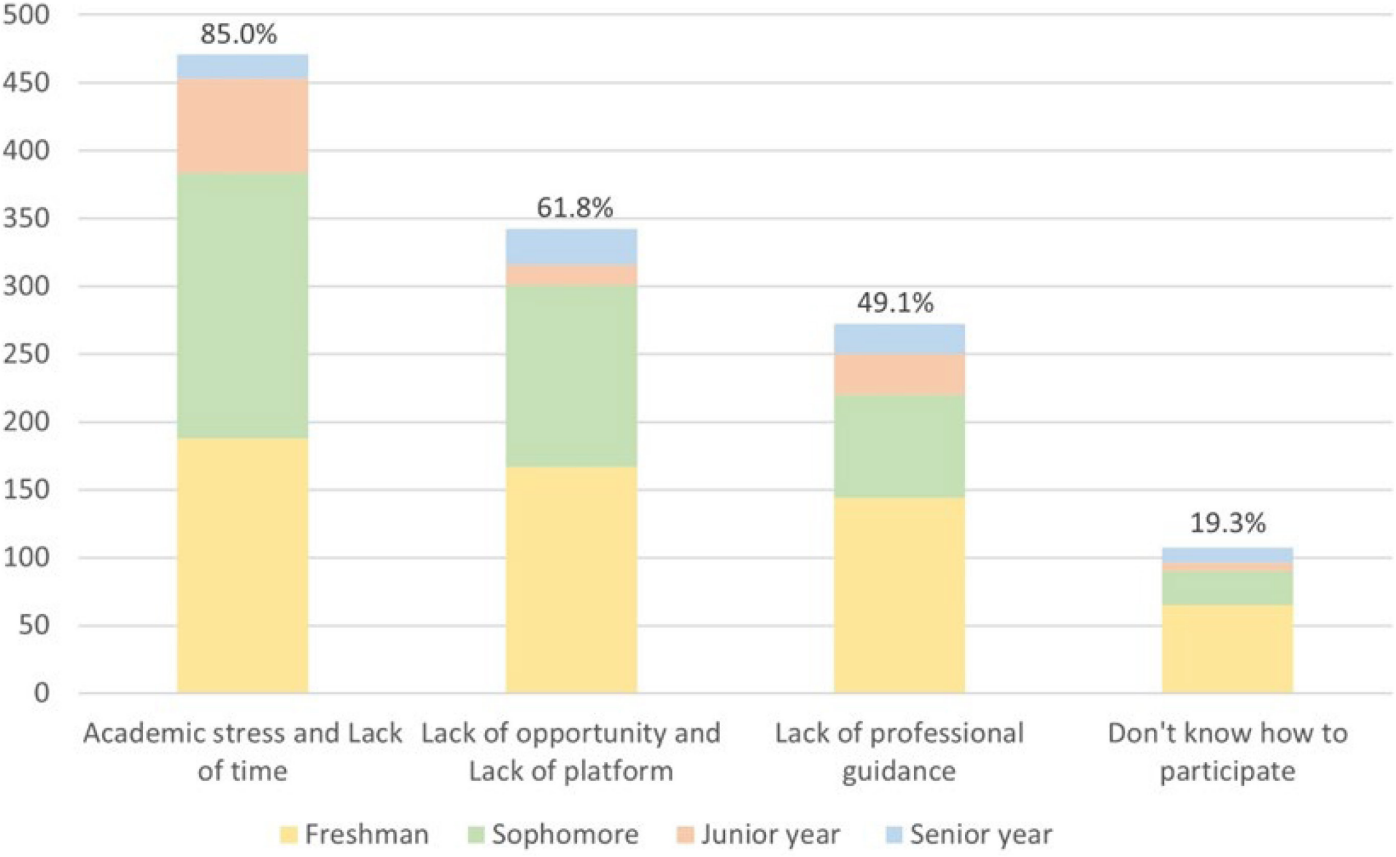
Factors influencing students' participation in scientific research in different grades. Scale = Number of people selecting this option/Total number of people 553.

**FIGURE 2:**
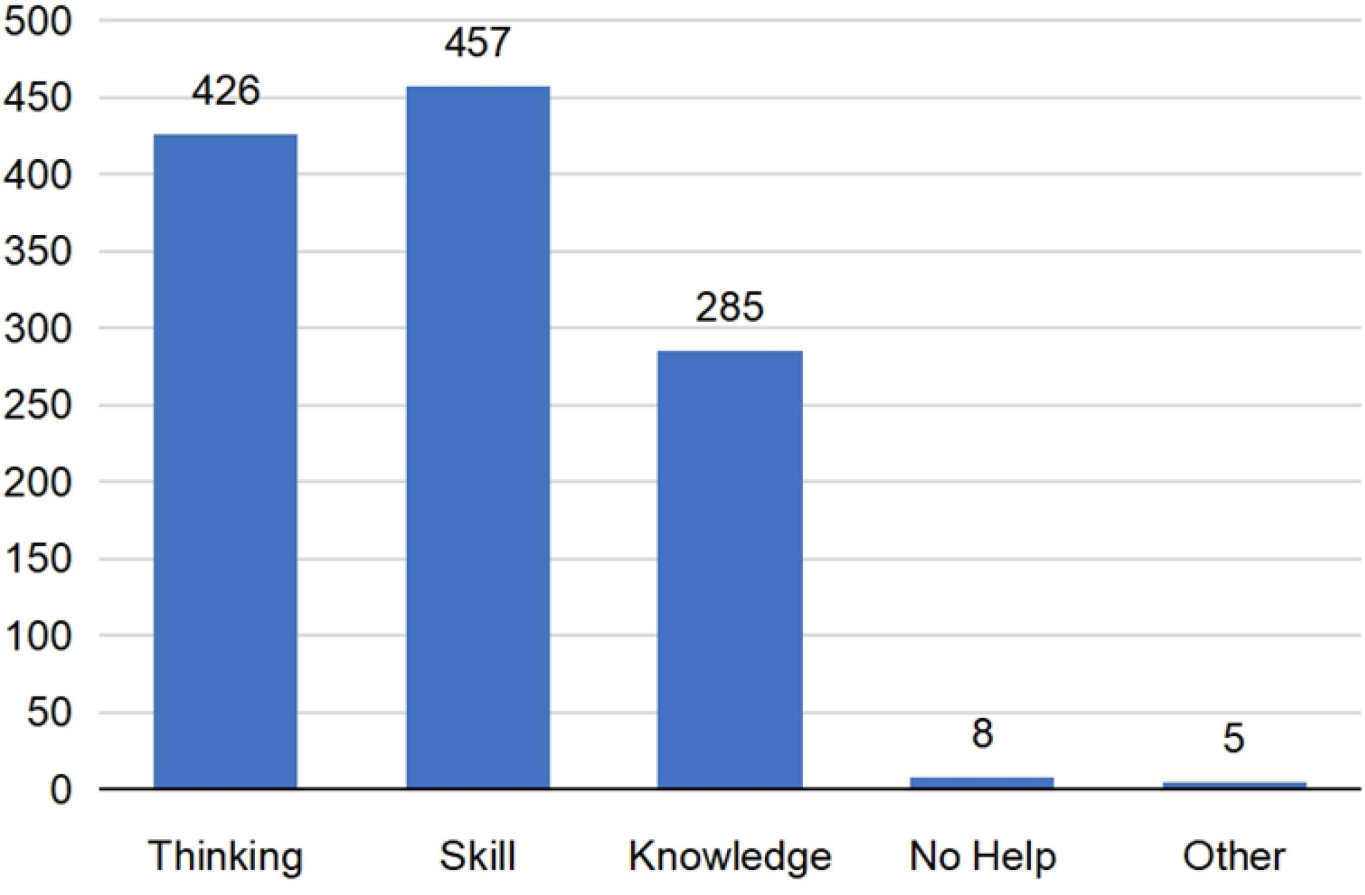
Aspects that students expect tutors to guide them.

The survey covered four major aspects: the basic situation of undergraduates' participation in scientific research, the main factors influencing their participation in scientific research, the type of support students want from their mentors, and ways to improve their scientific literacy. The survey items were in various forms, including multiple‐choice, multi‐select, and open‐ended questions, and they covered a wide range of topics. The questions addressed in Tables 1 and 2 are single‐choice, and the questions addressed in Figures 1 and 2 are multiple‐choice. For each question, we used Excel 2021 Microsoft to collect, organize, and analyze the information. The survey, a paper questionnaire, was completed anonymously with all respondents' prior informed consent. The returned questionnaires were strictly screened, and all invalid responses were excluded.

**TABLE 1:** Survey results of students participating in scientific research. (553 students in total).

Name	Options	Percentage (%)
Interest in scientific experiments	Higher	340	61.5
	General	194	35.1
	Lower	19	3.4
Views on Undergraduates' participation in scientific research experiments	Very important	241	43.6
	More important	259	46.8
	General	51	9.2
	Unimportant	2	0.4
Participate in scientific research experiments	Often	97	17.5
	Occasionally	255	46.1
	Not involved	201	36.4

**TABLE 2:** Survey results of students' ability to read, sort out, and apply literature.

Name	Options	Frequency	Percentage (%)
Read professional literature	At least once per week	72	13.0
	At least once per month	193	34.9
	At least once per year	288	52.1
Ability to organize and use documents	Proficiency	36	6.5
	Mastered, but not proficient	290	52.4
	Not mastered	227	41.1

## 3 RESULTS

### 3.1 Questionnaire results

Five hundred and ninety‐four questionnaires were distributed and collected, of which 553 were valid, with a valid return rate of 93.1%. Although part of the problem has been confirmed by previous studies,^5^, ^6^, ^7^ this paper analyzes it in a different way, yielding experimental results that are not identical to those of previous studies.

### 3.2 Survey respondents' participation in scientific research

The survey results showed that 61.5% of the students had an intense interest in research experiments, and 43.6% thought it was important for undergraduates to participate in research experiments, but only 17.5% participated in research experiments often (Table 1). This indicates that most university/college students have recognized the importance of research practice in improving their overall ability as undergraduates but are still lacking in practical operation.

### 3.3 Factors that hinder survey respondents' participation in research experiments

The survey results showed that academic stress, lack of opportunities and platforms, and lack of professional guidance are the main factors that hinder students' participation in research experiments, among which academic stress was the most important factor, with a prevalence of 85.0%. (Figure 1).

### 3.4 Types of support that survey respondents want from their mentors

According to the survey results for multi‐select questions, most students hoped that their mentors would provide training in innovative thinking and practical research skills, and some indicated that they hoped their mentors would share theoretical knowledge (Figure 2).

### 3.5 Survey respondents' ability to read, organize, and use literature

The survey results showed that 52.1% of the students read literature at least once per year, and only 13.0% read literature at least once per week; moreover, 93.5% of the students were not proficient at organizing and using literature (Table 2). The data indicate that students' habit of reading literature needs development, as does their ability to organize and use literature.

## 4 DISCUSSION

To adapt to the fast‐developing era, colleges and universities in China have always taken improving undergraduates' scientific research quality and cultivating their research and innovation ability as the focus of teaching reform. With the deepening of the reform, undergraduates' scientific research ability has improved significantly, but there are still many aspects that need to be enhanced. By reporting the results of this survey on undergraduates' scientific research situation, the author offers the following suggestions regarding the current status of undergraduate scientific research and the problems that tend to arise.

### 4.1 Stimulate interest in scientific research and adopt a favorable attitude

The survey results showed that juniors' disinterest in scientific research was significantly greater than that of seniors, probably because (1) compared to seniors, freshmen and sophomores have little or no contact with scientific research experiments, and they are curious about scientific research experiments and have a more intense interest; and (2) juniors and seniors have relatively clear career plans and are facing pressures such as postgraduate entrance examinations, graduation, and employment; hence, they do not have sufficient time to participate in research practice. Consequently, we believe that high‐quality undergraduate scientific research should be cultivated as early as possible. Teachers can update related teaching content in real‐time through new media channels in juniors' theoretical classes to broaden students' scientific knowledge. Furthermore, colleges and universities can offer a variety of independent experimental courses from which students can choose according to their interests. In these courses, students can design experiments, subject to instructors' modification and approval. Students can then manage and complete the experiments independently. While experimenting, students may develop the ability to discover problems on their initiative, think independently, and use their knowledge flexibly to answer questions. The university or department can also invite senior scholars or experts from home and abroad to deliver lectures and conduct “lecture hall” activities to introduce students to the latest academic developments and scientific achievements in professional research, answer student's questions about the profession, and generally guide students, with the aims of piquing their interest and stimulating their creativity.^8^ Through participation in research experiments, students are exposed to the research process and may master basic experimental techniques, which will, in turn, help them to develop the ability to identify and solve problems, as well as adopt a favorable attitude toward research. Overall, stimulating undergraduates' interest in scientific research and guiding their active and appropriate participation in scientific research will improve their overall ability and pave the way for success in postgraduate entrance examinations and at work.

### 4.2 Reasonable scheduling to facilitate students' exploration of scientific research knowledge

The survey found that the most important factor that prevented the survey respondents from participating in scientific research experiments was academic stress. Some differences in the proportion of students who selected academic stress were observed according to the year of study. “Based on a survey of our undergraduate students' research and a review of relevant literature,^7^, ^9^, ^10^ we found that among freshmen, 90.4% chose academic stress, probably because at that initial level of tertiary study, students have not yet fully adapted to the pace of university studies and have not implemented a reasonable schedule because of the sudden transition from stressful high school life to relaxed university life. According to the actual situation of our school, we came up with the following results that the reason sophomores (91.2%) chose academic stress may be the large increase in the number of major courses and the need to prepare for CET 4 and 6, which can lead to a general perception of academic stress among sophomores. Through the literature review,^10^ we found that seniors perceive academic stress as high, with the reason juniors (87.3%) chose academic stress may be that they had to face a series of examinations, such as postgraduate entrance examinations, public examinations, and teacher qualification exams, which greatly reduced their time for research. Few seniors (33.3%) chose academic stress because seniors have completed their major courses and their thesis projects and are mostly engaged in research experiments. Our data reflect problems that are common in Chinese universities, so our findings are somewhat credible. Meanwhile, there are still limitations because the undergraduate research mentorship system has been implemented in our college for many years and has achieved significant results, and students' interest and motivation in research have increased significantly. The system can stimulate students' interest in research more, so some students have adapted to the university rhythm quickly with the support of the system, and their pressure‐bearing ability has been exercised and they actively participate in experiments. Therefore this group of students did not choose the option of academic pressure. Considering all the factors, undergraduates generally think that the most important obstacle to their participation in research experiments is academic stress. Notably, there is a common phenomenon in Chinese colleges and universities where the proportion of theoretical courses far exceeds that of research experimentation courses, resulting in students having insufficient time to gain an in‐depth understanding of experiments.^11^ To address this problem, schools can appropriately increase the proportion of experimental courses according to the characteristics of the profession. Additionally, students can take the initiative to shadow teachers and seniors to conduct scientific research experiments in their spare time, to receive professional guidance, broaden their horizons, and improve their scientific literacy. During the experimental process, students should promptly communicate with their teachers and classmates when they encounter problems they do not understand, to deepen their understanding of the experiments and gain a more comprehensive grasp of the scientific research experimentation process. A study on undergraduate research experimentation revealed that some students, although they had been involved in scientific research for a long time, did not understand the principles of certain experimental steps, and only knew one side of the story. This suggests that some students cannot think deeply and also lack the spirit of exploration to get to the bottom of the problem. Students should think carefully about the meaning and role of each experimental step when doing experiments, cherish every opportunity to participate in experiments, and strive to gain something from each experiment.

### 4.3 Improve the project‐based undergraduate research mentorship system

Undergraduate research mentorship is a significant model for cultivating practical skills, scientific literacy, and innovative thinking in undergraduates. The careful guidance of mentors plays a key role throughout students' scientific research process.^12^ Many colleges and universities actively explore the undergraduate research mentorship system for talent cultivation,^13^ and this model has greatly contributed to the undergraduate talent cultivation mechanism and management system. Among the options, project‐based undergraduate research mentorship is more conducive to increasing opportunities for students to interact with their mentors. We investigated the students' understanding of the undergraduate scientific research tutorial system and compared it with the research results of relevant literature. We found that the results are similar to the published research results, because most of the respondents are not familiar with the undergraduate scientific research tutorial system and are eager to learn more information. But there are also differences. For example, the published research results pointed out that most of the respondents believed that the undergraduate scientific research tutorial system would help to formulate the future scientific research capacity development plan; our research results show that most of the respondents believe that the undergraduate scientific research tutorial system is helpful to cultivate scientific research innovative thinking and experimental skills. The project‐based undergraduate scientific research tutorial system can fully meet the wishes of students. Thus, universities and departments should build project platforms to fully mobilize students' enthusiasm and initiative. Driven by the project background, students can benefit from sufficient communication with their mentors and broaden their knowledge reserve.^14^ During this period, students not only master necessary skills but also consolidate their knowledge of professional courses, laying the foundation for comprehensive talent. Compared with traditional education methods, project‐based mentorship is more motivating and better guides students to do more meaningful research in their spare time, instead of indulging in web surfing. Ultimately, in a project‐based mentorship, a good academic atmosphere will develop among research groups, which is conducive to the development of students' scientific literacy.

### 4.4 Emphasis on data collection and organization and other abilities

Undergraduates engaged in scientific research tend to focus on the application of experimental skills, but many lack the ability to read literature, collect and organize scientific research data, and write papers.^15^ According to the survey, 13.0% of students read literature at least once per week; of these, 98.0% were juniors or seniors. Furthermore, 6.5% of students, all of whom were juniors or seniors, claimed to have mastered the ability to organize and use literature. It can be inferred that juniors and seniors already possess basic scientific literacy gained through systematic cultivation and application. However, since juniors and seniors only accounted for 23.5% of the total number of survey respondents, which is far less than the percentage of freshmen and sophomores, and considering that freshmen and sophomores have not yet acquired the habit of reading literature or mastered the ability to organize and use literature, the final data revealed that most students read literature less frequently and have not mastered the skills to apply the knowledge that literature imparts. Therefore, the data are more representative of lower‐grade students. Further, the data showed that 52.1% of students read literature at least once per year, and 93.5% were not proficient at organizing and using literature. University students need to form the habit of reading literature and improve their ability to organize and apply it. To address these problems, mentors can recommend excellent literature and supervise students' regular perusal of core journals. Additionally, universities/colleges can host literature discussions to deepen students' knowledge of research. Most importantly, students should take initiative, correct their learning attitude, and be self‐motivated to read literature regularly.^16^ Moreover, students should seek to solve the problems they encounter in regular experiments by consulting the literature to advance their understanding of the experiments and their mastery of the literature. Students can broaden their horizons and enrich their knowledge base by collecting, organizing, and summarizing relevant literature and data. Through specialized training from schools and mentors, students can sharpen their information collection and paper writing, and defense skills and improve themselves, their comprehensive ability, and their scientific literacy, thereby laying the foundation for developing into sources of innovative scientific talent in the future.

In conclusion, the cultivation of high‐quality undergraduate scientific research requires the cooperation of many parties and constant in‐depth research. The country should strengthen the system for cultivating innovative talent in scientific research, universities should explore the establishment of suitable platforms for cultivating high‐quality undergraduate scientific research, and teachers should participate in the scientific research mentorship system for undergraduates. Considering their career plans, undergraduates should combine their professional knowledge with theory and practice, take initiative, refine their comprehensive scientific research quality, lay a solid foundation for scientific research, and take their own unique scientific research path. Only by acquiring solid research skills can aspiring undergraduates stand out in their future research and develop high‐quality research talent.

## AUTHOR CONTRIBUTIONS

*Conception and design*: Jing Luan. *Date collection and analysis*: Ling Liu. *Manuscripit writing and final approval*: All authors

## DISCLOSURE

The authors have no conflicts of interest with respect to this study.

## ETHICS APPROVAL STATEMENT

The Ethics Review Committee approved the study. (No: 2022‐21).

## Supporting information


Data S1:

## Data Availability

The data that support the findings of this study are available from the corresponding author upon reasonable request.
