# Supplementary material for: Survey analysis and discussion on cultivating scientific research quality among undergraduates in medical colleges
Source: Pharmacol Res Perspect. 2023 May 19;11(3):e01095. doi: 10.1002/prp2.1095 (PMC10197879; doi:10.1002/prp2.1095)
Supplement: Supplementary file 1 — Data S1: [file PRP2-11-e01095-s001.docx]

**Questionnaire for the cultivation of students' research quality**

Hello! Thank you very much for taking time out of your busy schedule to complete this survey on the quality of student research. The purpose of this survey is to understand the research situation of undergraduate students and to discuss rationalization measures to further improve the quality of undergraduate research.

This questionnaire management for anonymous survey, only for research, your personal information and privacy we will strictly confidential, please rest assured, thank you very much.

1. [fill in the blank] *

College: _______________ Major: _______________

Grade: _______________ Gender: _______________

2. Interest in scientific experiments [Single-choice] *

| ○Higher |
| --- |
| ○General |
| ○Lower |

3. Perceptions of undergraduate students' participation in research experiments [Single-choice] *

| ○Very important |
| --- |
| ○More important |
| ○General |
| ○Not important |

4. Factors that prevent you from participating in scientific experiments [multiple choice] *

| □Academic stress and lack of time |
| --- |
| □ Lack of opportunity, lack of platform |
| □ Lack of professional guidance |
| □ Don't know how to participate |
|  |

5. Are you aware of the undergraduate research mentorship [multiple choice] *

| ○Very well understood |
| --- |
| ○General |
| ○Do not understand |

6. In what areas you would like your mentor to help [multiple choice] *

| □ Innovative thinking in research |
| --- |
| □ Hands-on practical skills |
| □ Theoretical knowledge |
| □ No help needed |
| □ Other _________________ |

7. Participation in scientific research experiments [Single-choice] *

| ○Often |
| --- |
| ○Occasionally |
| ○No involved |

8. Reading professional literature [multiple choice] *

| ○At least once per week |
| --- |
| ○At least once per month |
| ○At least once per year |

9. The ability to organize and apply the literature [Single-choice] *

| ○Proficiency |
| --- |
| ○Mastered, but not proficient |
| ○Not mastered |

10. How good is your English [Multiple Choice] *

| ○CET4 |
| --- |
| ○CET6 |
| ○IELTS |
| ○TOEFL |
| ○Other _________________ |

11. Do you think you will be engaged in scientific research in the future? If not, please explain [fill in the blank] *

_________________________________
